# An Autopsy Case of Severe COVID-19 Pneumonia Complicated by Intrapulmonary Thrombosis in Myelodysplastic/Myeloproliferative Neoplasm With Ring Sideroblasts and Thrombocytosis

**DOI:** 10.7759/cureus.62790

**Published:** 2024-06-20

**Authors:** Yoshinori Harada, Masahiro Makino, Ryuta Nakao, Yuji Shimura, Takehiro Ogata, Michiyo Hayakawa, Hirokazu Shiraishi, Junya Kuroda, Satoaki Matoba, Hideo Tanaka

**Affiliations:** 1 Department of Pathology and Cell Regulation, Graduate School of Medical Science, Kyoto Prefectural University of Medicine, Kyoto, JPN; 2 Department of Cardiovascular Medicine, Graduate School of Medical Science, Kyoto Prefectural University of Medicine, Kyoto, JPN; 3 Division of Hematology and Oncology, Department of Medicine, Graduate School of Medical Science, Kyoto Prefectural University of Medicine, Kyoto, JPN

**Keywords:** myelodysplastic/myeloproliferative neoplasm, thrombocytosis, ring sideroblasts, pulmonary thrombosis, autopsy, pneumonia, covid-19

## Abstract

Patients with coronavirus disease 2019 (COVID-19) pneumonia are prone to intrapulmonary thrombosis owing to excessive inflammation and platelet activation. Myelodysplastic/myeloproliferative neoplasm (MDS/MPN) with ring sideroblasts and thrombocytosis (RS-T) is a rare disease in MDS/MPN overlap entities. Patients with MDS/MPN RS-T are known to be at a high risk of thrombosis, and platelet count control with drug therapy does not necessarily reduce this risk. Here, we report the autopsy case of an older male patient with MDS/MPN RS-T and severe COVID-19 pneumonia complicated by intrapulmonary thrombosis. His platelet count had been controlled in the normal range after treatment with hydroxyurea and 5-aza-2'-deoxycytidine. On admission day, he rapidly developed respiratory distress and tested positive on a polymerase chain reaction test for severe acute respiratory syndrome coronavirus-2 (SARS-CoV-2). After admission, he received supplemental oxygen and was administered remdesivir and dexamethasone; however, his respiratory and circulatory status did not improve. The patient died on day 4 of illness. Autopsy findings revealed massive thrombi within blood vessels and diffuse alveolar damage in both lungs, which were determined to be the cause of death. In patients with MDS/MPN RS-T combined with COVID-19 pneumonia, clinicians may need to pay close attention to the risk of pulmonary thrombosis.

## Introduction

Coronavirus disease 2019 (COVID-19), caused by severe acute respiratory syndrome coronavirus-2 (SARS-CoV-2), began to spread worldwide in December 2019, leading to the death of approximately seven million people as of June 2023 [1,2]. On May 5, 2023, the World Health Organization stated that COVID-19 no longer constituted a public health emergency of international concern [3]. However, SARS-CoV-2 continued to mutate. The main causes of death related to COVID-19 infection are acute respiratory distress syndrome and intrapulmonary thrombosis [4-6].

Here, we report the autopsy of an older male patient with COVID-19 pneumonia and myelodysplastic/myeloproliferative neoplasm (MDS/MPN) with ring sideroblasts and thrombocytosis (RS-T) [7]. The patient rapidly developed massive intrapulmonary thrombosis and diffuse alveolar damage (DAD) after SARS-CoV-2 infection. MDS/MPN RS-T is a subtype of MDS/MPN characterized by the presence of thrombocytosis (≥45×10^4^/μL), <1% blasts in the peripheral blood, RS accounting for ≥15% of erythroblasts, dyserythropoiesis, and <5% blasts in the bone marrow [7]. Patients with MDS/MPN RS-T are known to be at a high risk of thrombosis. To our knowledge, this is the first report of COVID-19 pneumonia complicated by intrapulmonary thrombosis in MDS/MPN RS-T. In patients with COVID-19 pneumonia who are at high risk for thrombosis, such as in our case, clinicians may need to pay careful attention to the risk of pulmonary thrombosis.

## Case presentation

Clinical summary

**Figure 1:**
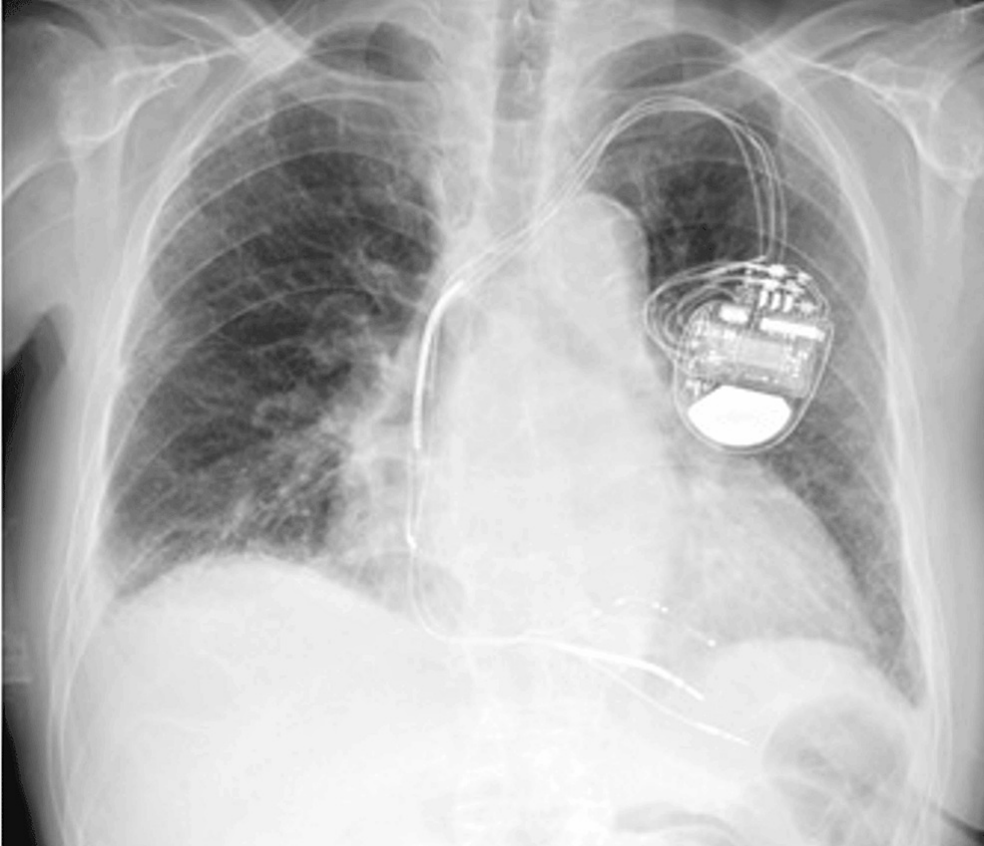
Chest radiography results before the onset of COVID-19 pneumonia

A man in his early 80s presented to our hospital with respiratory distress. Fifteen years previously, he had been admitted to an educational hospital for diabetes mellitus, which had been well-controlled since then. Two years later, he was diagnosed with heart failure with reduced left ventricular ejection fraction, chronic kidney disease, hypertension, and variant angina pectoris. He also received cardiac resynchronization therapy (CRT) for the treatment of heart failure with a complete left bundle branch block, as shown in Figure 1. His heart failure was effectively controlled after CRT implantation. He underwent partial hepatic resection a year later for hepatocellular carcinoma (HCC) in non-alcoholic steatohepatitis.

**Figure 2:**
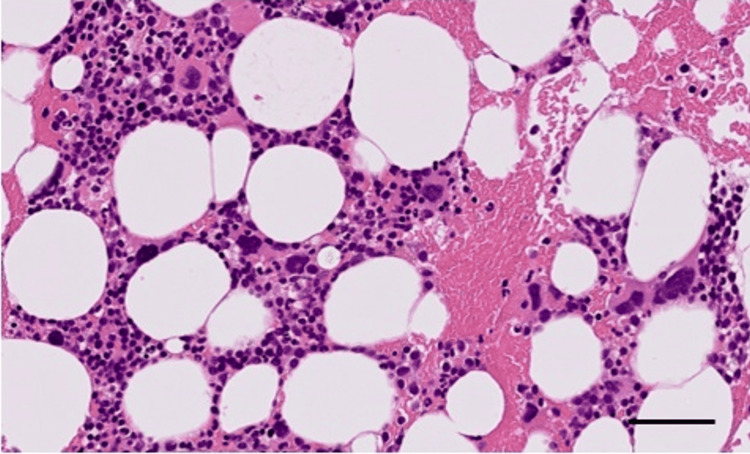
H&E staining of the bone marrow clot image at the onset of MDS/MPN RS-T. Scale bar = 50 µm MDS/MPN RS-T: myelodysplastic/myeloproliferative neoplasm with ring sideroblasts and thrombocytosis; H&E: hematoxylin and eosin

**Figure 3:**
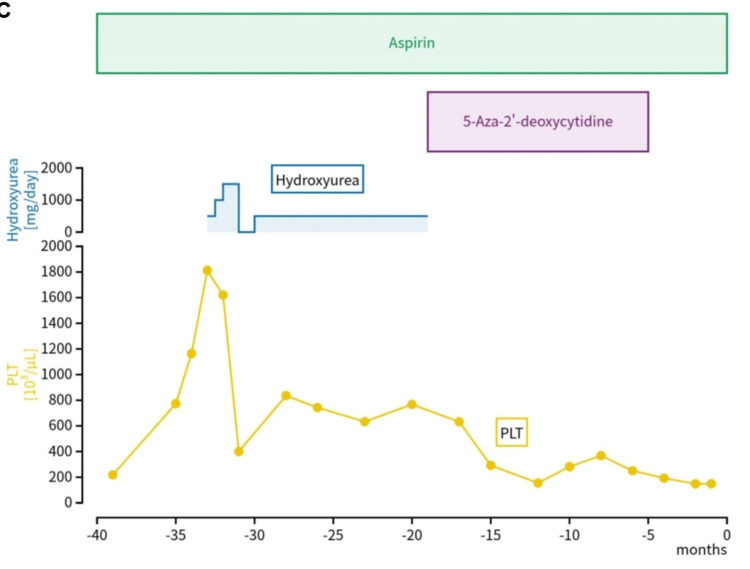
Clinical course showing the platelet count (PLT) (103 /µL) with the drugs administered

Three years before the current visit, he developed persistent anemia and thrombocytosis and was diagnosed with MDS/MPN RS-T without a JAK2 mutation or reduced von Willebrand factor activity. Figure 2 shows a magnified image of the bone marrow clot at the time of diagnosis, with a markedly increased megakaryocyte count of 10-30 per high-power field. The hematopoietic cell density was approximately 20% hypoplastic bone marrow. He received oral aspirin, hydroxyurea, and 5-aza-2'-deoxycytidine for thrombocytosis (Figure 3) and erythropoietin and blood transfusion (400 mL/week) for the treatment of severe anemia. His platelet count was controlled in the normal range after treatment with hydroxyurea and 5-aza-2'-deoxycytidine.

**Figure 4:**
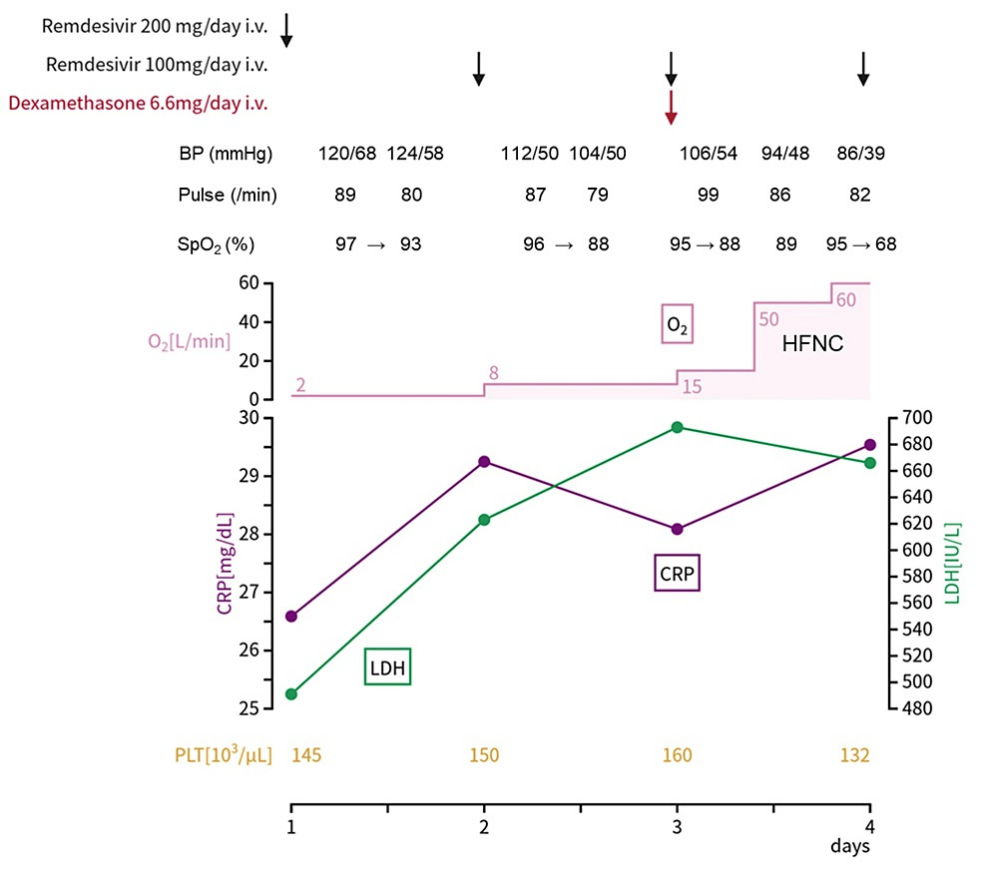
Clinical course after the onset of COVID-19 pneumonia showing the serum levels of CRP (mg/dL) and LDH (U/L), the amount of oxygen administered, and platelet count (103 /µL), along with the drugs administered CRP: C-reactive protein; LDH: lactate dehydrogenase; SpO2: percutaneous oxygen saturation; BP: blood pressure

On the day of the current admission, the patient rapidly developed respiratory distress and chest pain on inspiration and tested positive on a polymerase chain reaction (PCR) test for SARS-CoV-2. His systolic and diastolic blood pressure were 118/72 mmHg, pulse rate of 89 beats/minute, body temperature of 38.6 °C, and percutaneous oxygen saturation in ambient air at the time of admission 88%. He could be classified as obese as per his body mass index, which was 27.0. Table 1 shows the laboratory data on admission. The serum levels of lactate dehydrogenase (LDH) and C-reactive protein (CRP) gradually increased over time (Figure 4).

**Table 1:** Summary of the laboratory data at the time of admission

Variables	Result	Reference range
Peripheral blood		
White blood cells (×10^2^/μL)	208	32-85
Neutrophils (%)	81.5	45.2-68.8
Hemoglobin (g/dL)	8.2	13.1-17.6
Platelets (×10^4^/μL)	14.5	15.8-34.8
Blood biochemistry		
Total bilirubin (mg/dL)	0.53	0.1-1.2
Aspartate aminotransferase (U/L)	36	12-35
Alanine aminotransferase (U/L)	54	6-33
Lactate dehydrogenase (U/L)	491	114-243
γ-glutamyl transpeptidase (U/L)	59	3-54
Alkaline phosphatase (U/L)	60	120-362
Blood-urea-nitrogen (mg/dL)	41.6	7.0-20.0
Creatinine (mg/dL)	1.30	0.30-1.10
Total protein (g/dL)	6.2	6.4-8.3
Albumin (g/dL)	2.6	3.8-5.2
Sodium (mmol/L)	130	138-150
Potassium (mmol/L)	4.4	3.6-5.0
Calcium (mg/dL)	8.4	8.6-10.5
Glucose (mg/dL)	99	65-110
C-reactive protein (mg/dL)	26.59	0.00-0.20

**Figure 5:**
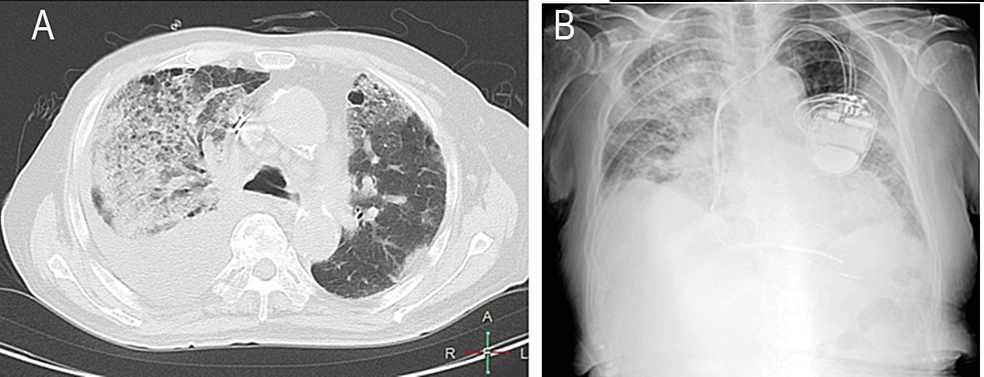
(A) Chest computed tomography on day 1; (B) Chest radiography on day 2

After admission, the patient received supplemental oxygen at a rate of 2 L/min via a nasal cannula and prophylactic therapy using foot pumps against deep venous thrombosis. His chest computed tomography results on admission (Figure 5A) showed numerous infiltrative shadows in both lungs suggesting pneumonia. The patient demonstrated progressive cardiac enlargement and dilatation of the inferior vena cava, indicative of potential heart failure. There was notable pleural effusion, predominantly on the right side. The patient then received remdesivir intravenously at a 200-mg loading dose on day 1, followed by a 100-mg maintenance dose on days 2-4. He was administered tazobactam/piperacillin, dobutamine, and furosemide to treat pneumonia and chronic heart failure. His respiratory and general conditions rapidly worsened on day 2, resulting in administration of supplemental oxygen at 8 L/min. Chest radiography on day 2 showed massive infiltrative shadows, mainly in the right lung, and enlargement of the cardiac silhouette (Figure 5B).

On day 3, 6.6 mg of dexamethasone was administered; however, his respiratory status did not improve. The blood clotting test results on day 3 of admission were as follows: prothrombin time-international normalized ratio 1.43 (reference range (RR), 0.89-1.12); activated partial thromboplastin time 42.9 seconds (RR, 23.6-31.3 sec), fibrinogen, 528 mg/dL (RR, 200-400 mg/dL), and fibrin degradation products 11.5 μg/mL (RR, <5 μg/mL). Due to renal dysfunction, contrast-enhanced computed tomography for the evaluation of intrapulmonary thrombosis was not performed. The patient received high-flow nasal cannula therapy with 100% oxygen delivery at a flow rate of up to 60 L/minute; however, the respiratory and circulatory status did not improve. The patient died on day 4 of illness.

Pathological findings

**Figure 6:**
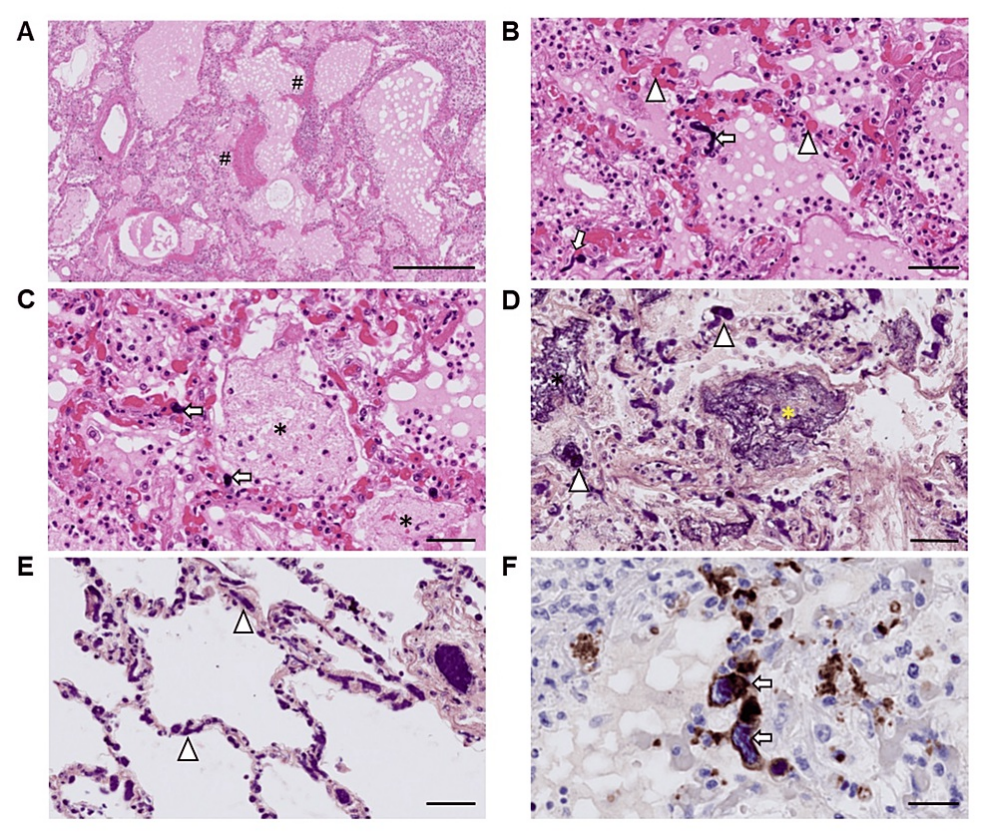
Microscopic findings of the autopsied lungs. (A) H&E-staining image (medium-power view) of the left upper lobe of the lung. Hyalin membrane formation is indicated by # signs. Scale bar = 500 µm; (B, C) H&E-staining images (high-power view) of the left upper lobe of the lung. The arrowheads, arrows, and asterisks show thrombi, megakaryocytic cells, and fibrin balls, respectively. Scale bars = 50 µm; (D) PTAH-staining image of the left upper lobe of the lung (high-power view). The arrowheads and asterisks show fibrin thrombi and a fibrin ball, respectively. Scale bar = 50 µm; (E) PTAH-staining image of the right lower lobe of the lung (high-power view). The arrowheads show fibrin thrombi; (F) Immunolabeling with anti-CD61, H&E counterstain. Immunohistochemistry reveals intrapulmonary megakaryocytes (arrowheads). Scale bar = 25 µm. H&E: hematoxylin and eosin; PTAH: phosphotungstic acid hematoxylin

**Figure 7:**
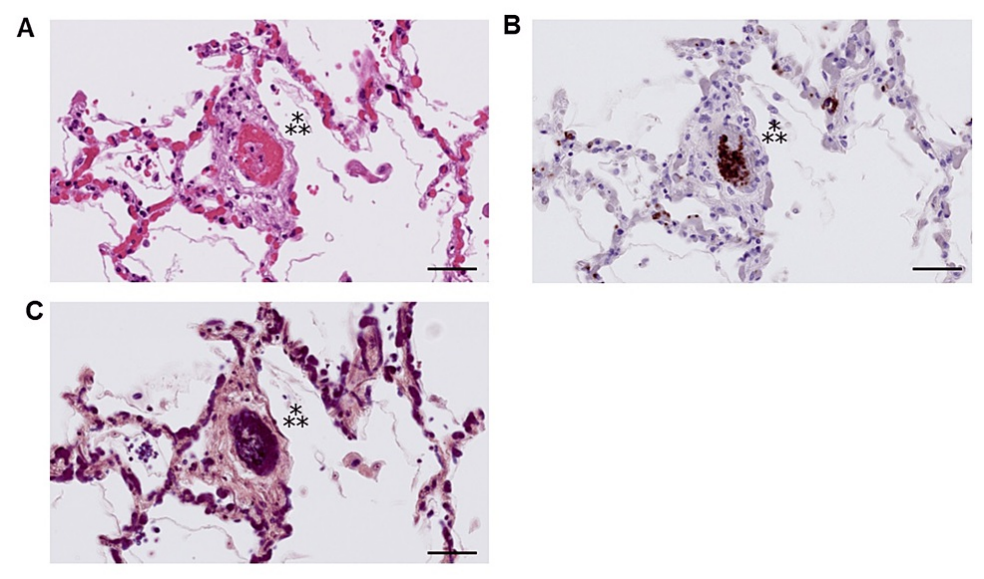
Serial tissue section analysis of the autopsied lung. (A) Hematoxylin and eosin-staining image, (B) CD61 immunostaining image of the right lower lobe, (C) Phosphotungstic acid hematoxylin-staining image.

An autopsy was performed three hours after death to precisely reveal the pathogenesis of the respiratory condition’s rapid deterioration. Increased bilateral lung weights (left lobes: 730 g, right lobes: 880 g) with congestion and edema were noted; bilateral pleural effusion (left: 100 mL, right: 900 mL) was also observed. Histologically, DAD was found in the exudative phase with hyaline membrane formation (Figure 6A). Macrophages, lymphocytes, and neutrophils were observed within the alveoli, and edema, congestion, intracapillary fibrin thrombi, and cells with large nuclei were seen (Figure 6B). Fibrin balls were observed within the alveolar space as fibrin-containing exudates (Figure 6C, 6D). Intravascular fibrin microthrombi were conspicuous even in areas without prominent edema and inflammation (Figure 6E). Hematoxylin and CD61-staining revealed intrapulmonary megakaryocytes with hyperpigmented nuclei and CD61-positive cytoplasm (Figure 6F). Bone marrow clots showed slightly hypercellular bone marrow with increased megakaryocytes (50 megakaryocytes per 8 HPF (high-power fields)), and monotonous proliferation of blast cells was not noted. Although the thrombi in the lung tissues consisted mostly of fibrin (Figures 6D, 6E), platelet-fibrin thrombi were also observed in a small portion of the lungs (Figure 7). We did not find an apparent sign of an intravascular large thrombus. Pulmonary thrombosis and DAD associated with SARS-CoV-2 infection were the main causes of respiratory and circulatory failure.

The patient’s heart weight increased to 570 g, and left ventricular hypertrophy was noted. A CRT defibrillator was implanted. Interstitial fibrosis, thought to be caused by essential hypertension, was also found predominantly on the endocardial side of the left ventricle. Right ventricular dilatation, inflammatory cell infiltration, or intravascular thrombosis was not observed. A small number of microthrombi were seen in the glomerular vessels of the kidney; however, there was no evidence of diabetic nephropathy. Hepatic congestion with sinusoidal dilatation around the central vein was observed. No apparent fatty deposits were noted in the hepatocytes. Berlin blue staining revealed marked iron deposition, suggesting hemosiderosis caused by multiple blood transfusions. There was no evidence of recurrence of HCC. The aorta exhibited atheroma, ulceration, and calcification, indicating atherosclerosis. No apparent abnormalities were observed in the esophagus, stomach, large intestine, or pancreas.

## Discussion

In this case, the patient died four days after the onset of symptoms. Histologically, the lungs showed extensive hyaline membrane formation and edema, suggesting an early phase of DAD. In addition, massive thrombi within blood vessels were observed, and respiratory and circulatory failure due to pulmonary thrombosis and DAD constituted the main cause of death. Pulmonary thrombosis and DAD are reported to be relatively typical findings of COVID-19 pneumonia [4,5].

A recent National Institutes of Health guideline recommends either remdesivir alone or dual administration of remdesivir and dexamethasone for hospitalized patients requiring conventional oxygen [8]. In particular, remdesivir without dexamethasone is recommended for patients with COVID-19 infection requiring only minimal oxygen [8]. This patient required supplemental oxygen at a rate of 2 L/minute via a nasal cannula on admission, and we initially administered remdesivir. However, after admission, the patient's symptoms progressed rapidly and we were unable to confirm a clear effect of remdesivir, so dexamethasone was administered on the third day of admission.

The patient had MDS/MPN RS-T for three years. Although most of the thrombi in the lung tissue comprised fibrin thrombi, fibrin platelet thrombi were also observed. Patients with MDS/MPN RS-T are known to be at high risk for thrombosis [9]; in patients with essential thrombocythemia (ET), a disease analogous to MDS/MPN RS-T, controlling platelet counts with pharmacotherapy does not necessarily result in a lower incidence of thrombosis [10-12]. Takasaki et al. reported COVID-19 pneumonia associated with ET [13], which can cause thrombosis associated with COVID-19. Thus, we could not exclude the possibility that the pathophysiology of MDS/MPN RS-T influenced thrombus formation in this case.

Many megakaryocytes were found in the lungs in the present case. Megakaryocytes are usually found predominantly in the bone marrow. However, extramedullary megakaryocytes are frequently found in the microvasculature of the lungs in COVID-19 pneumonia [14-16]. The presence of a large number of intrapulmonary megakaryocytes may influence thrombus formation; however, further studies on the relationship between intrapulmonary megakaryocytes and thrombosis are needed.

The pathogenesis of thrombosis in patients with COVID-19 is undetermined but is likely related to platelet overactivation [17]. Infection of megakaryocytes and platelets by SARS-CoV-2 has been reported as a cause of platelet activation [17-19]. Incubation of plasma from patients with severe COVID-19 with platelets from healthy volunteers increased the level of platelet activation markers (P-selectin and CD63) [17,20]. It has also been reported that an immune complex composed of COVID-19 spike protein and related antibodies may contribute to platelet activation and thrombosis [17,21].

We did not administer anticoagulant heparin to this patient. A recent guideline does not recommend therapeutic-dose low-molecular-weight heparin or unfractionated heparin in critically ill hospitalized patients [22]. However, in the case of non-critically ill patients hospitalized for COVID-19, the use of prophylactic-dose low-molecular-weight heparin or unfractionated heparin is beneficial [22-25]. Antithrombotic therapy for COVID-19 is expected to evolve in the future with an increase in clinical studies.

## Conclusions

This report described the autopsy case of an older male patient with MDS/MPN RS-T, who developed intrapulmonary thrombosis and DAD after being infected with SARS-CoV-2. It was considered that the clinical characteristics of hypercoagulability and immune dysregulation of MDS/MPN RS-T synergistically exacerbated COVID-19 pneumonia. COVID-19 infection may increase the probability of thrombosis in MDS/MPN RS-T with controlled platelet counts. Further studies are needed to establish safe and effective treatment strategies for this condition.
